# Amebicidal Activity of *Escherichia coli* Nissle 1917 Against *Entamoeba histolytica*

**DOI:** 10.3390/microorganisms13040828

**Published:** 2025-04-05

**Authors:** Vivian Moura-Oliveira, Fabrício M. S. Oliveira, Olga L. M. Moreno, Julia R. Ferreira, Raphael E. Szawka, Ana C. Campideli-Santana, Jullia Teles, Luciano S. A. Capettini, Flaviano S. Martins, Maria A. Gomes

**Affiliations:** 1 Department of Parasitology, Federal University of Minas Gerais, Belo Horizonte 31270-901, MG, Brazil; vivian-moura@live.com (V.M.-O.); juliarodriguesferreira.16@gmail.com (J.R.F.); juju54321@live.com (J.T.); 2 Translational Type 2 Immunity Laboratory, Microbiology, Immunology and Tropical Medicine Department, George Washington School of Medicine and Health Sciences, Washington, DC 20052, USA; oliveirafms13@gmail.com; 3 Department of Pharmacology, Federal University of Minas Gerais, Belo Horizonte 31270-901, MG, Brazil; olgamore2@gmail.com (O.L.M.M.); lucianocapettini@gmail.com (L.S.A.C.); 4 Department of Physiology and Biophysics, Federal University of Minas Gerais, Belo Horizonte 31270-901, MG, Brazil; reszawka@gmail.com (R.E.S.); anaclaracampideli@gmail.com (A.C.C.-S.); 5 Department of Microbiology, Federal University of Minas Gerais, Belo Horizonte 31270-901, MG, Brazil; flaviano@icb.ufmg.br

**Keywords:** *Entamoeba histolytica*, *Escherichia coli*, Nissle, probiotic, reactive oxygen species

## Abstract

Amebiasis is a globally prevalent infection that can lead to fatal outcomes if not adequately treated. Conventional treatment with imidazoles often fails due to side effects and resistance, emphasizing the need for alternative therapies. The probiotic *Escherichia coli* Nissle 1917 (EcN) has shown potential in combating intestinal pathogens. This study aimed to evaluate the amebicidal activity of EcN in vitro and its effect on the production of reactive oxygen species (ROS). Trophozoites of *Entamoeba histolytica* (2.5 × 10⁴ cells/mL) were cultured in 96-well plates and exposed to varying concentrations of EcN (10^2^–10^9^ cells/mL). Plates were incubated at 36 °C for 6, 12, and 18 h, after which trophozoite viability was assessed. Intracellular ROS production, including superoxide and hydrogen peroxide, was measured using fluorescent probes. The highest efficacy was observed after 18 h at a CFU concentration of 10^9^ cells/mL. Increased ROS production at all probiotic concentrations suggested a role in EcN’s amebicidal mechanism. Morphological changes in trophozoites, such as rounding, vacuolization, and size reduction, were noted after EcN exposure, indicating growth inhibition. These findings suggest EcN induces structural and morphological changes in *E. histolytica*, inhibiting its growth in vitro. The findings suggest the potential efficacy of EcN; however, definitive confirmation requires data from human clinical trials.

## 1. Introduction

*Entamoeba histolytica* is an anaerobic eukaryotic protozoan and the etiological agent of amebiasis. The infection is globally distributed, with a higher prevalence in developing countries, particularly in regions with inadequate water treatment. There is a notable incidence in areas where contaminated water containing cysts, the infective form of the parasite, is used for vegetable cultivation [1,2].

Infection with *E. histolytica* can present as symptomatic or asymptomatic [3]. The primary site of infection is the intestine; however, the amoeba can disseminate to other organs, resulting in extraintestinal amebiasis, with the liver being the most common site. In developing countries, diarrhea remains the third leading cause of mortality in children under five years of age [4], and amebiasis is listed among the top 15 causes of mortality [5].

An estimated 50 million cases of invasive *E. histolytica* infection occur annually, resulting in approximately 100,000 deaths. The prevalence of infection exhibits global variation, ranging from 10% to 50% across different regions worldwide [6,7]. However, it is believed that this percentage may be underestimated, as asymptomatic infections can be caused by both *E. histolytica* and *E. dispar*, which are morphologically indistinguishable.

The treatment of choice for amebiasis is metronidazole [8]. However, in up to 50% of patients, this medication alone fails to eradicate intestinal colonization, leaving affected individuals at a substantial risk of relapse months later. This peculiarity underscores the necessity for research into alternative therapeutic options that, in addition to ensuring safety, demonstrate effective activity while minimizing adverse effects and resistance. In this context, probiotics emerge as promising alternatives for either curative or preventive treatment [9,10,11].

A well-known probiotic is *Escherichia coli* Nissle 1917 (EcN) [12,13,14]. EcN is a Gram-negative enterobacterium that can colonize the intestine within a few days and persist as a colonic microbiota for months after administration [15,16]. This interaction of EcN with the microbiota promotes direct antagonistic effects, such as the inhibition of the growth and death of pathogenic bacteria and yeasts. Indirect antagonistic effects involve the inhibition of the invasion of intestinal epithelial cells by invasive pathogens and include signaling with the intestinal mucosa [17]. EcN inhibited the growth of 21 bacterial isolates, including 40% for *Pseudomonas*, 50% for *E. coli*, *Enterococcus*, and *Staphylococcus*, and 100% for *Klebsiella* and *Enterobacter* [18]. EcN has also been shown to effectively inhibit the adhesion of the adherent-invasive *E. coli* isolated from Crohn’s disease patients, supporting its use as an adjunctive treatment for these patients [19]. There is limited research evaluating the effects of EcN in parasitic infections. The therapeutic potential of this probiotic in amebiasis has not yet been assessed. Therefore, the objective of our study was to expand research on probiotics for the treatment of amebiasis by assessing the amebicidal activity of EcN in vitro and its effect on the production of reactive oxygen species.

## 2. Materials and Methods

### 2.1. E. histolytica Strain

The axenic strain EGG of *E. histolytica* used in this study was isolated in our laboratory from a patient residing in Manaus, Amazonas, who presented with dysentery. The patient’s diagnosis was confirmed through serology, zymodeme analysis, and PCR, all of which were positive for *E. histolytica* [20].

The strain was maintained in axenic culture and grown in TYI-S-33 medium [21]. Inoculations were performed every 72–96 h, and the tubes were stored with an inclination of 30 degrees in a bacteriological oven under temperature of 37 °C.

### 2.2. Escherichia coli Nissle 1917

The probiotic EcN (Mutaflor; Ardeypharm, Herdecke, Germany) was acquired as a pharmaceutical product available in Canada. EcN was isolated and maintained in brain-heart infusion broth under aerobic conditions for 24 h at 37 °C. Subculturing was performed every 24 h to ensure its use during the exponential growth phase.

EcN was acclimated in TYI-S-33 medium prior to association with *E. histolytica* to ensure its viability during the assays. Colony-forming units (CFUs) were determined through serial dilution in TYI-S-33 medium to achieve concentrations ranging from 10^2^ to 10^9^ cells/mL. Culture viability, colony counts, and purity were assessed via Gram staining for quality control.

### 2.3. In Vitro Association

Initially, 40,000 *E. histolytica* trophozoites were distributed into a 96-well plate. The plate was then incubated at 37 °C for 1 h to ensure adherence of the trophozoites to the plate surface. After the incubation, the supernatant was discarded. To the wells containing *E. histolytica*, 270 µL of TYI-S-33 medium and 30 µL of *E. coli* Nissle (EcN) were added in serial dilutions, achieving concentrations ranging from 10^2^ to 10^9^ cells/mL. The plate was subsequently incubated with the probiotic concentrations at 37 °C for 6, 12, and 18 h. Following each incubation period, the viability of the amoebae was assessed by counting using a hemocytometer and trypan blue exclusion.

The wells containing only *E. histolytica*, without the addition of EcN, were considered as controls, with 100% viable cells. The number of viable trophozoites for each probiotic concentration was quantified and compared to the control to calculate the percentage of inhibition at different association times and concentrations.

The final inhibition percentage for each treatment was calculated using the following formula adapted from Edington [22]: I (%) = [(CEhC − CEhT)/CEhC] × 100, where I represents the inhibition percentage; CEhC denotes the trophozoite growth of *Entamoeba histolytica* in the control; and CEhT indicates the trophozoite growth of *Entamoeba histolytica* in the treatment.

### 2.4. Morphometric Analysis of the Trophozoites

For the morphometric analysis of trophozoite size, the area of each trophozoite was measured, considering that live trophozoites could assume an amoeboid shape. Images of 15 viable trophozoites and 15 dead trophozoites were captured using a JVC TK-1270/RGB microcamera (Tokyo, Japan) under a 40× objective for digitization. The area occupied by each trophozoite was measured using QuPath software version 0.5.1 (https://qupath.github.io, accessed on 19 February 2025).

### 2.5. Determination of Reactive Oxygen Species

To assess intracellular reactive oxygen species (ROS) production, specifically superoxide radicals and hydrogen peroxide, intracellular fluorescent probes were employed. Following the treatments, the cells were washed with PBS (pH 7.2) and subsequently incubated with fluorescent probes for superoxide (dihydroethidium, DHE, 5 µM; Invitrogen, Waltham, MA, USA) and hydrogen peroxide (dihydrodichlorofluorescein diacetate, H2-DCF-DA, 5 µM; Invitrogen, USA) for 30 min. The DAPI probe was used for nuclear staining (for 5 min). All procedures were conducted in the dark, and cells were washed with PBS after staining. Post-incubation, the cells were fixed with 4% paraformaldehyde and subsequently examined using fluorescence microscopy for further analysis.

### 2.6. Fluorescence

Initially, round cover slips of 13 mm diameter were placed into each well of a 24-well plate. Subsequently, 200 µL of a solution containing 40,000 trophozoites of *E. histolytica* were distributed into each well. To ensure the adherence of the amoebae to the glass cover slips, the plate was incubated at 37 °C for 1 h. Following incubation, the supernatant was discarded. In wells containing *E. histolytica*, 270 µL of TYI-S-33 medium and 30 µL of probiotic bacteria, previously diluted to achieve successive dilutions ranging from 10^4^ to 10^9^ CFU, were added. The plate was incubated again at 37 °C for 6, 12, and 18 h. No antibiotics were employed during the co-cultivation experiment. Finally, the cover slips were removed from the plate and mounted on slides for imaging under a fluorescence microscope, with emission/excitation wavelengths set at 525/470 nm and 629/572 nm for H2-DCF-DA and DHE probes, respectively.

After the incubation period, the plates were examined using an inverted microscope to assess cell growth, viability, and adherence. Images were captured with a 20× objective on a fluorescence microscope (Axiovision 3.1, Zeiss, Hallbergmoos, Germany). Immunoreactive cells were identified by their green cytoplasm and blue nuclei. Photographs of fields containing 5 to 10 cells were taken for each experimental treatment across three fluorescent channels—blue, green, and red.

### 2.7. Statistical Analysis

In the in vitro assay, a two-way ANOVA was conducted to compare the effects of different *E. coli* Nissle concentrations across different time points (6 h, 12 h, and 18 h). To identify the specific differences in viability at each time point, Tukey’s post-hoc test for multiple comparisons was employed (*p* < 0.05). Data were processed using the statistical software package GraphPad Prism, version 9.0.

## 3. Results

### 3.1. Effect of Probiotic EcN on E. histolytica Viability

**Figure 1:**
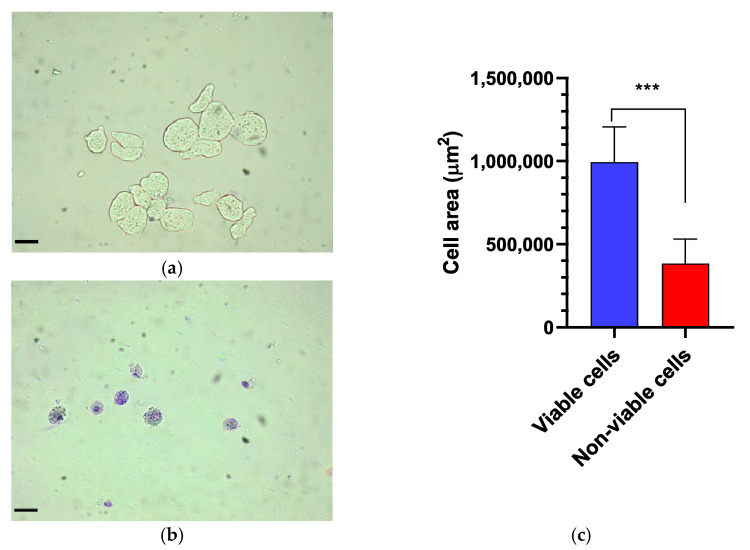
Morphological assessment of Entamoeba histolytica trophozoites. (**a**) Control. Viable trophozoites exhibit a larger size and remain unstained by trypan blue. (**b**) In contrast, non-viable trophozoites display a rounded shape, increased cytoplasmic granularity, and intense staining with trypan blue. (**c**) Comparison of the trophozoite cell area between viable and non-viable cells, with a significant difference (*p* < 0.0007). Bar = 70 µm. *** *p* < 0.001.

**Figure 2:**
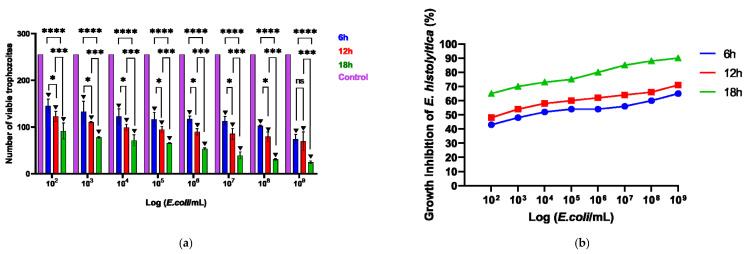
(**a**) Number of viable cells following the association of the parasite *E. histolytica* with the probiotic *E. coli* Nissle, subjected to different concentrations (10^2^–10^9^) across various time intervals, in a 96-well plate. (**b**) Impact of probiotics on the inhibition of *E. histolytica* trophozoites over different time periods and at varying concentrations. * *p* < 0.05; *** *p* < 0.01; **** *p* < 0.001; ▼ *p* < 0.001 Significant difference when compared to the control group.

The viability of *E. histolytica* following association with EcN was assessed by counting viable trophozoites using a hemocytometer at intervals of 6, 12, and 18 h. Following exposure to the probiotic, the dead or distressed trophozoites, observed under an optical microscope, appeared stained with trypan blue and generally had a rounded shape (Figure 1a,b). Additionally, they were smaller in size compared to viable trophozoites, as confirmed by measuring the area occupied by each trophozoite (Figure 1c). The number of trophozoites was quantified and graphically represented in relation to the variation in EcN concentration (Figure 2a). Inhibition of *E. histolytica* growth was observed at all incubation times and across all probiotic concentrations. This inhibitory effect was most evident after 18 h of association at a CFU of 10^9^ cells/mL (Figure 2b).

### 3.2. ROS Production

The production of superoxide and hydrogen peroxide was evaluated in the co-culture of EcN and *E. histolytica*. We focused on the analysis of ROS production at 6 h and 18 h. These were the two extremes of our experimental design and showed significant differences in the number of trophozoites between them.

**Figure 3:**
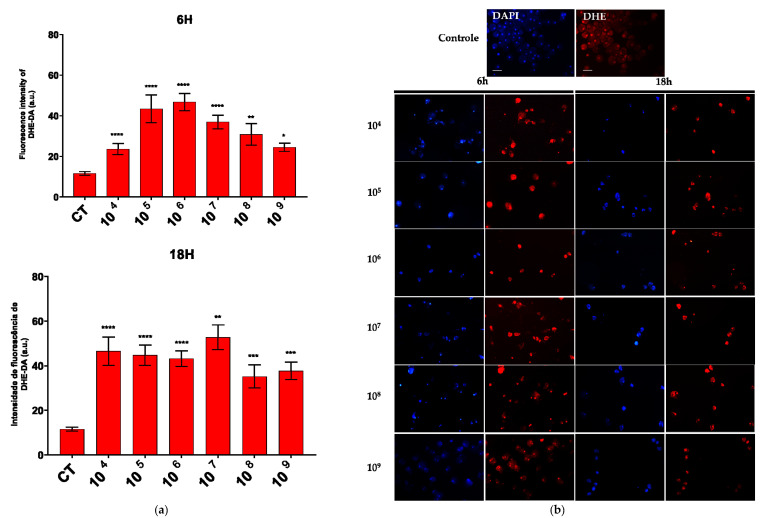
ROS production evaluated by DHE and DCF fluorescence. (**a**) Quantification of DHE–DA fluorescence intensity in each group. (**b**) Fluorescence microscopic images of intracellular production of DHE–DA staining in *E. histolytica* after interaction with the probiotic *E. coli* Nissle at different concentrations over 6 h and 18 h. Bar = 50 µm. * *p* < 0.05; ** *p* < 0.002; *** *p* < 0.0005; **** *p* < 0.0001.

An increase in the production of both compounds was observed across all the associated time points and probiotic concentrations. Notably, a marked enhancement in superoxide production was detected at a concentration of 10^6^ cells/mL after six hours of association and at 10^7^ cells/mL after eighteen hours (Figure 3a).

**Figure 4:**
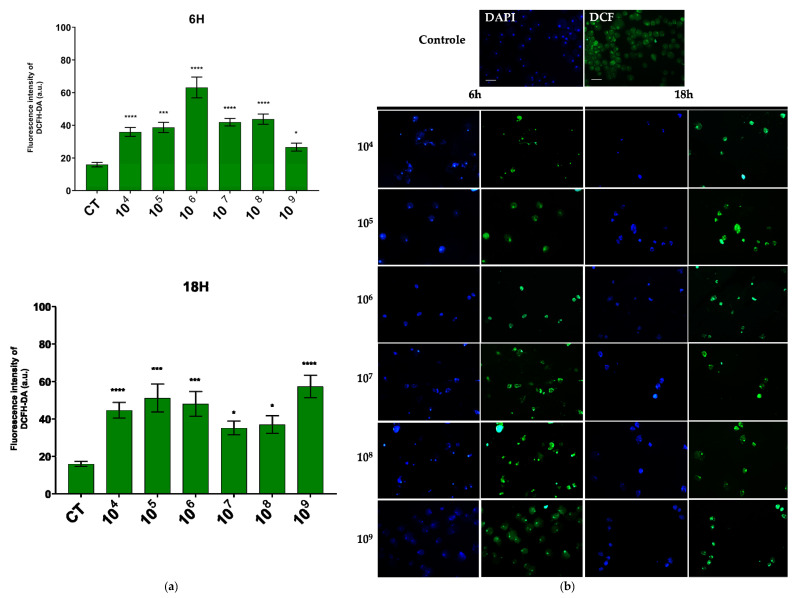
ROS production evaluated by DCFH–DA fluorescence. (**a**) Quantification of DCFH–DA fluorescence intensity in each group. (**b**) Fluorescence microscopic images of intracellular production of DCFH–DA staining in *E. histolytica* after interaction with the probiotic *E. coli* Nissle at different concentrations over 6 h and 18 h. Bar = 50 µm. * *p* < 0.004; *** *p* < 0.0003; **** *p* < 0.0001.

Regarding hydrogen peroxide, the concentration that showed the most pronounced reaction was also 10^6^ cells/mL over a 6-h period, while in the 18-h period, the concentration of 10^9^ cells/mL was most effective (Figure 4a).

In the fluorescence analysis, a clear morphological alteration of the trophozoites was observed following their interaction with the probiotic. The cells acquired a more rounded shape and exhibited vacuolation, accompanied by a reduction in size. Additionally, there was an apparent loss of the intracellular ameboid medium (Figure 3b and Figure 4b).

## 4. Discussion

The treatment of choice for amebiasis involves adverse effects that often lead to the discontinuation of therapy. Additionally, the suspicion of drug resistance signals the importance of alternative therapies in resolving the infection. Probiotics have emerged as potentially attractive options. However, the use of probiotics has been timidly evaluated in amebiasis. The combination of *Saccharomyces boulardii* and metronidazole for the treatment of amebiasis has been reported to reduce the duration of clinical symptoms and cyst excretion [23]. Furthermore, *S. boulardii* has demonstrated effectiveness in inhibiting the adherence of amoebae to the intestinal mucosal surface [24]. *Lactobacillus helveticus* has been identified as a potential probiotic for amebiasis [25,26], while *Lactobacillus casei* and *Enterococcus faecium* have demonstrated in vitro activity against *E. invadens* [27]. However, the precise mechanism of action of these probiotics remains incompletely understood. Our research group assessed the efficacy of the lactic acid bacterium *Weissella paramesenteroides* WpK in resolving the lesions induced by *E. dispar* in murine models. The bacterium facilitated the recovery of necrotic regions by enhancing intestinal mucosal protection through the upregulation of MUC-2 and epithelial regeneration [28].

EcN is a well-established probiotic with recognized benefits for intestinal homeostasis [29,30,31]. In this context, we investigated the potential of EcN to contribute to the control of *E. histolytica* infection. Our results demonstrated a reduction in the proliferation of *E. histolytica* trophozoites in the presence of EcN, suggesting an anti-amebic activity of the probiotic. This activity was time-dependent, with maximum efficacy observed at 18 h, resulting in over 80% inhibition of *E. histolytica* trophozoite growth. These findings support the therapeutic potential of EcN in the treatment of amebiasis.

In addition to the relationship between exposure time and the ability of EcN to inhibit the growth of *E. histolytica*, the quantity of probiotic cells is also crucial for the effectiveness of its activity. Inhibition of *E. invadens* growth was observed at a concentration of 10^8^ CFU/mL, with *E. faecium* demonstrating approximately 71% inhibition of the parasite’s growth, and *L. casei* showing approximately 50% inhibition [27].

In this study, the most effective treatment was observed at a concentration of 10^9^ CFU/mL of EcN. The efficacy of a probiotic in vivo depends on the specific strain, but generally, the effective dosage for inducing favorable changes in the intestinal microbiota ranges from 10^8^ to 10^9^ CFU/mL [32]. Although this study was conducted in vitro, the findings indicate that probiotic concentration plays a crucial role in the observed effects, aligning with dosages recommended in the in vivo studies. In vitro models are valuable tools for investigating initial mechanisms and providing insights for future in vivo research. Notably, all the concentrations of EcN tested resulted in a reduction in *E. histolytica* proliferation, reinforcing its potential therapeutic application in amoebiasis.

When associating EcN with *E. histolytica*, a clear reduction in the proliferation of the amoebas was observed. Anaerobic microorganisms possess inherently weak antioxidant defenses against oxidative stress. In *E. histolytica*, ROS act as significant cytotoxic effectors, causing protein oxidation which generally leads to the inhibition of protein synthesis [33]. Probiotics may serve as effective allies in controlling the proliferation of pathogenic microorganisms through the production of ROS.

In the association between EcN and *E. histolytica*, the production of hydrogen peroxide and superoxide was observed. The generation of free radicals is a common process during interactions between microorganisms. The probiotic effect of *Lactobacillus helveticus* has been attributed to its capacity to produce hydrogen peroxide [25,34].

*E. histolytica* trophozoites exhibited a more rounded and vacuolated morphology, along with a reduction in size following association with EcN. Additionally, a loss of intracellular amebic content was observed. Collectively, these findings indicate that EcN acts as a causative agent of the morphological and structural alterations observed in the amebic cells.

Among the potential mechanisms of action identified for the microbicidal activity of EcN are the enhancement of transepithelial resistance [35], a beneficial effect on the formation and stabilization of epithelial tight junctions [36], the positive regulation of zona occludens-1 mRNA expression [37], the induction of human β-defensin 2, an inducible antimicrobial peptide synthesized by the epithelium to counteract bacterial adhesion and invasion [38], and a protective role against pathogenic *E. coli* strains that colonize the intestines of patients with inflammatory bowel disease [19]. For the first time, the possibility of probiotic activity of EcN via reactive oxygen species is suggested. The production of hydrogen peroxide and superoxide by EcN may represent a mechanism through which the probiotic exerts its amebicidal activity.

## 5. Conclusions

The results of this study provide good evidence that the probiotic EcN inhibits the growth of *E. histolytica* trophozoites. This inhibitory effect was accompanied by significant morphological changes in the trophozoites, including rounding, vacuolization, and reduction in size. A notable decrease in trophozoite numbers was observed in cultures exposed to EcN, with the most pronounced effect occurring at a concentration of 10^9^ cells/mL and after an 18-h incubation period, suggesting a dose- and time-dependent relationship. Additionally, for the first time, this study identifies the production of hydrogen peroxide and superoxide as a novel mechanism of action for EcN, which likely contributes to its inhibitory effects. Together, these findings shed new light on the therapeutic potential of EcN in the treatment of amoebiasis and encourage further research to explore the underlying mechanisms involved.

## Data Availability

The data that support the findings of this study are available in the article. Further inquiries can be directed to the corresponding author.
